# Leaf variegation caused by plastome structural variation: an example from *Dianella tasmanica*

**DOI:** 10.1093/hr/uhae009

**Published:** 2024-01-10

**Authors:** Shuaixi Zhou, Kainan Ma, Jeffrey P Mower, Ying Liu, Renchao Zhou

**Affiliations:** State Key Laboratory of Biocontrol and Guangdong Provincial Key Laboratory of Plant Resources, School of Life Sciences, Sun Yat-sen University, Guangzhou 510275, China; State Key Laboratory of Biocontrol and Guangdong Provincial Key Laboratory of Plant Resources, School of Life Sciences, Sun Yat-sen University, Guangzhou 510275, China; Center for Plant Science Innovation and Department of Agronomy and Horticulture, University of Nebraska, Lincoln, NE 68588, USA; State Key Laboratory of Biocontrol and Guangdong Provincial Key Laboratory of Plant Resources, School of Life Sciences, Sun Yat-sen University, Guangzhou 510275, China; State Key Laboratory of Biocontrol and Guangdong Provincial Key Laboratory of Plant Resources, School of Life Sciences, Sun Yat-sen University, Guangzhou 510275, China

## Abstract

Variegated plants often exhibit plastomic heteroplasmy due to single-nucleotide mutations or small insertions/deletions in their albino sectors. Here, however, we identified a plastome structural variation in albino sectors of the variegated plant *Dianella tasmanica* (Asphodelaceae), a perennial herbaceous plant widely cultivated as an ornamental in tropical Asia. This structural variation, caused by intermolecular recombination mediated by an 11-bp inverted repeat flanking a 92-bp segment in the large single-copy region (LSC), generates a giant plastome (228 878 bp) with the largest inverted repeat of 105 226 bp and the smallest LSC of 92 bp known in land plants. It also generates an ~7-kb deletion on the boundary of the LSC, which eliminates three protein coding genes (*psbA*, *matK*, and *rps16*) and one tRNA gene (*trnK*)*.* Albino sectors exhibit dramatic changes in expression of many plastid genes, including negligible expression of *psbA*, *matK*, and *rps16*, reduced expression of photosynthesis-related genes, and increased expression of genes related to the translational apparatus. Microscopic and ultrastructure observations showed that albino tissues were present in both green and albino sectors of the variegated individuals, and chloroplasts were poorly developed in the mesophyll cells of the albino tissues of the variegated individuals. These poorly developed chloroplasts likely carry the large and rearranged plastome, which is likely responsible for the loss of photosynthesis and albinism in the leaf margins. Considering that short repeats are relatively common in plant plastomes and that photosynthesis is not necessary for albino sectors, structural variation of this kind may not be rare in the plastomes of variegated plants.

## Introduction

Plastids are vital for photosynthesis and the biosynthesis of starch, fatty acids, amino acids, and pigments in plants [91]. Plastid genomes (plastomes) of most angiosperms are typically 120–160 kb in size and organized in a quadripartite structure, including a large single-copy region (LSC), a small single-copy region (SSC), and two inverted repeats (IRs) [63, 69, 75]. Different copies of plastid DNA are usually thought to be homogenous in sequence in the cells of a plant; however, intra-individual heteroplasmy has been observed in many plants, including rice [62], cotton [49], *Pinus* [21, 93], *Medicago* [15, 34, 51], *Oenothera* [9], *Actinidia* [8], and *Citrus* [6]. Plastid heteroplasmy is generally attributed to mutations [23, 44] or biparental inheritance [6, 8, 15]. Heteroplasmy in plastome structure was also detected in some plants, including *Eleocharis* [50], *Monsonia* [76], *Sciadopitys* [33], *Taxus* [17], and Cupressoideae [26, 73]. These alternative plastome conformations are usually caused by repeat-mediated intramolecular recombination.

Variegation, which manifests as differently colored (green or albino) sectors on vegetative or reproductive organs of a plant [44], can arise by mutations in the plastid, mitochondrial, or nuclear genomes [23, 44, 74, 77, 86, 99]. However, variegated plants are a focus of plastome heteroplasmy studies because green sectors often contain cells with normal chloroplasts, while albino sectors contain cells with abnormal plastids [23]. Spontaneous mutations in the plastomes have been identified in albino mutants and variegated plants, such as *Antirrhinum* [79], *Cryptomeria japonica* [32], barley [47, 48], sunflower [2], *Oenothera* [19, 60, 95], and many others [70]. These spontaneous mutations are typically either single-nucleotide substitutions or small insertions/deletions of 1–50 bp, which can often cause loss of function of a plastid gene. Recently, a large deletion of 425 bp in *rpoC2* of *Clivia miniata* var. *variegata* was identified and suggested to contribute to its leaf variegation [97]. To date, however, no structural variation in plastomes arising from genome rearrangement has been reported in any variegated plant. Yet large-scale deletions have been detected in long-term cultured plant cell lines [39, 56] and albino plants regenerated by anther culture [12–14, 30, 31].

**Figure 1:**
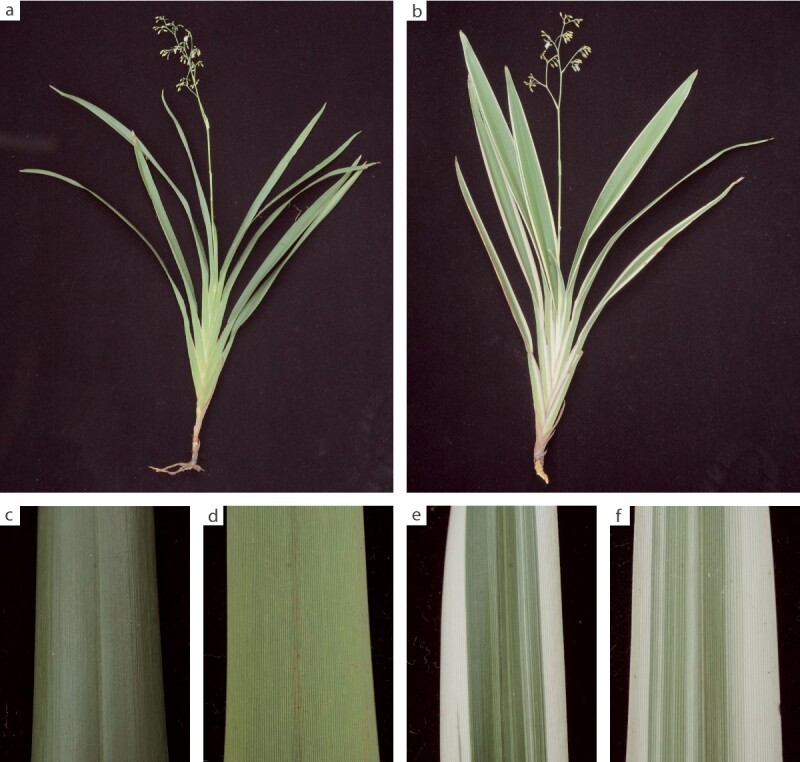
Photographs of green and variegated plants of *D. tasmanica*. **a** Green plant. **b** Variegated plant. **c** Adaxial leaf surface of a green plant. **d** Abaxial leaf surface of a green plant. **e** Adaxial leaf surface of a variegated plant. **f** Abaxial leaf surface of a variegated plant.

Here we identified a plastome structural variation in the albino sector of *Dianella tasmanica* (Asphodelaceae), a perennial herbaceous plant widely cultivated in tropical Asia for ornamental purposes (Fig. 1). This structural variation, mediated by an 11-bp IR, leads to a large, rearranged, and incomplete plastome, with dramatic changes in expression of many plastid genes in the albino sectors. In addition, we examined the microscopic characters of albino and green tissues in a light microscope and chloroplast ultrastructure of albino and green tissues in a transmission electron microscope (TEM), and found some anatomical changes in the variegated individuals, which may explain their plastomic heteroplasmy.

## Results

### Green *D. tasmanica* individual has a plastome typical of angiosperms

**Figure 2:**
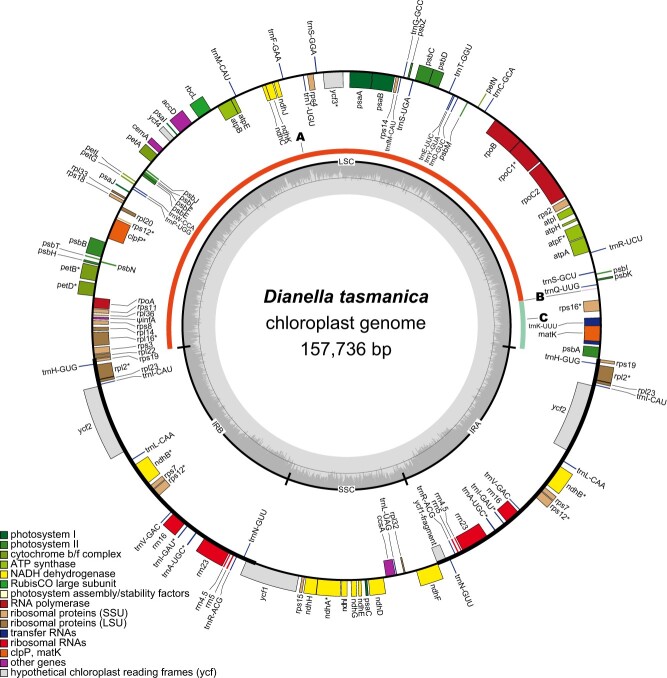
Gene map of the plastome of the green plant of *D. tasmanica*. Genes shown outside the outer circle are transcribed counterclockwise, whereas those inside the outer circle are transcribed clockwise. The dark and light shading inside the inner circle indicates GC and AT content, respectively. Asterisks (^*^) indicate genes containing intron(s). Areas A, B, and C, which are defined in the Results section, are shown between the inner and outer circles.

Using Illumina reads from the leaf tissue of a green individual of *D. tasmanica*, we assembled a circular molecule of 157 736 bp for its plastome (we designated it as a G-type plastome). The plastome possesses a typical quadripartite structure, including an LSC of 85 422 bp, an SSC of 18 334 bp, and a pair of IRs of 26 990 bp each. The overall GC content of this plastome is 37.5% (Table 1). The plastome encodes 109 unique genes, including 78 protein-coding, 4 rRNA, and 27 tRNA genes (Fig. 2, Table 1). *infA* was annotated as a pseudogene due to the presence of multiple in-frame stop codons. Pseudogenization or loss of *infA* has been found in many lineages of angiosperms [61]. The size, structure, and gene content of the plastome are all typical of most angiosperms.

**Table 1:** Summary of the G- and A-type plastomes in *D. tasmanica*.

**Features**	**G-type**	**A-type**
Genome size (bp)	157 736	228 878
LSC size (bp)	85 422	92
SSC size (bp)	18 334	18 334
IR size (bp)	26 990	105 226
Genome GC content (%)	37.5	37.0
LSC GC content (%)	35.4	31.7
SSC GC content (%)	31.7	31.5
IR GC content (%)	42.8	37.5
Number of total (unique) protein genes	85 (78)	138 (75)
Number of total (unique) rRNAs	8 (4)	8 (4)
Number of total (unique) tRNAs	35 (27)	51 (26)

### Albino sectors of variegated *D. tasmanica* individuals have a giant plastome with the largest inverted repeat and the smallest large single-copy region in land plants

For each of the two variegated individuals of *D. tasmanica*, we assembled the same circular molecule of 228 878 bp for the plastome of the albino sectors (we designated it as an A-type plastome). The size of the A-type plastome is 45% larger than that of the G-type plastome. It is among the largest land plant plastomes reported, only smaller than the plastomes of *Pelargonium transvaalense* (242 575 bp), *Vitis romanetii* (232 020 bp), and *Pelargonium endlicherianum* (230 012 bp), but larger than the *Pelargonium* × *hortorum* plastome (217 942 bp) [10, 92, 98]. The plastome has a quadripartite structure, containing a pair of greatly expanded IRs of 105 226 bp each, separated by an extremely contracted LSC of only 92 bp and an SSC of 18 334 bp. Note that we designated the LSC and SSC of the A-type plastome based on their homology to the G-type plastome rather than their length, and that the LSC is in fact much shorter than the SSC because of LSC contraction. This structure was confirmed by nanopore reads (Supplementary Data Fig. S1). The A-type IR is the longest one described to date from land plants, four times longer than those of most other angiosperms (~25 kb) [75] and exceeding the longest reported IRs from *P. transvaalense* (87 724 bp) [92] and *P.* × *hortorum* (75 741 bp) [10]. The A-type LSC, by contrast, was reduced to only ~0.1% the size compared with LSCs of most other angiosperms (80–90 kb), and is the shortest one in land plants yet sequenced. The overall GC content of the A-type plastome is 37.0%, which is slightly lower than that of the G-type plastome.

### Green sectors of variegated *D. tasmanica* individuals have two types of plastome

**Figure 3:**
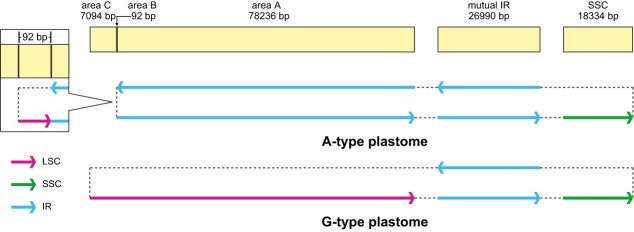
Structural difference between G- and A-type plastomes in *D. tasmanica*. Plastid sequences are shown as light yellow blocks, with sequence identity and their sizes shown on the top. The quadripartite structure and connections among the LSC, IRs, and SSC of G- and A-type plastomes are indicated by arrows and dashed lines. Like those of most other angiosperms, the G-type plastome is composed of the LSC, SSC, and two copies of the IR (shown in the middle of this figure). In the A-type plastome, area A is moved to the IR, resulting in a greatly expanded IR (26 990 + 78 236 bp) and a greatly contracted LSC (only 92 bp), and area C is deleted, while the SSC is unchanged relative to the G-type plastome (shown in the lower of this figure). Here the 92-bp area is enlarged for a clear view. See the Results section for definitions of G- and A-type plastomes and areas A, B, and C.

For each of the two variegated individuals of *D. tasmanica*, we assembled from the green sectors both an A-type plastome and a G-type plastome. By comparing the two plastomes, we found two major changes in the A-type plastome: a 78 236-bp region (area A) was moved from the LSC to the IR, resulting in a greatly expanded IR and reduction of the LSC, and the LSC was further reduced to only 92 bp (area B) by a deletion of 7094 bp (area C) on the boundary of the LSC of the G-type plastome (Fig. 3). The rearrangement around the LSC (the A–B–A structure) was confirmed by both Illumina and nanopore reads (Supplementary Data Fig. S2) and the ~7-kb deletion was confirmed by nanopore reads (Supplementary Data Fig. S3). The deleted area C contains three protein-coding genes, namely, *psbA*, *matK*, and *rps16*, and one tRNA gene, *trnK*.

### Intermolecular recombination mediated by a pair of 11-bp inverted repeats produces the A-type plastome

**Figure 4:**
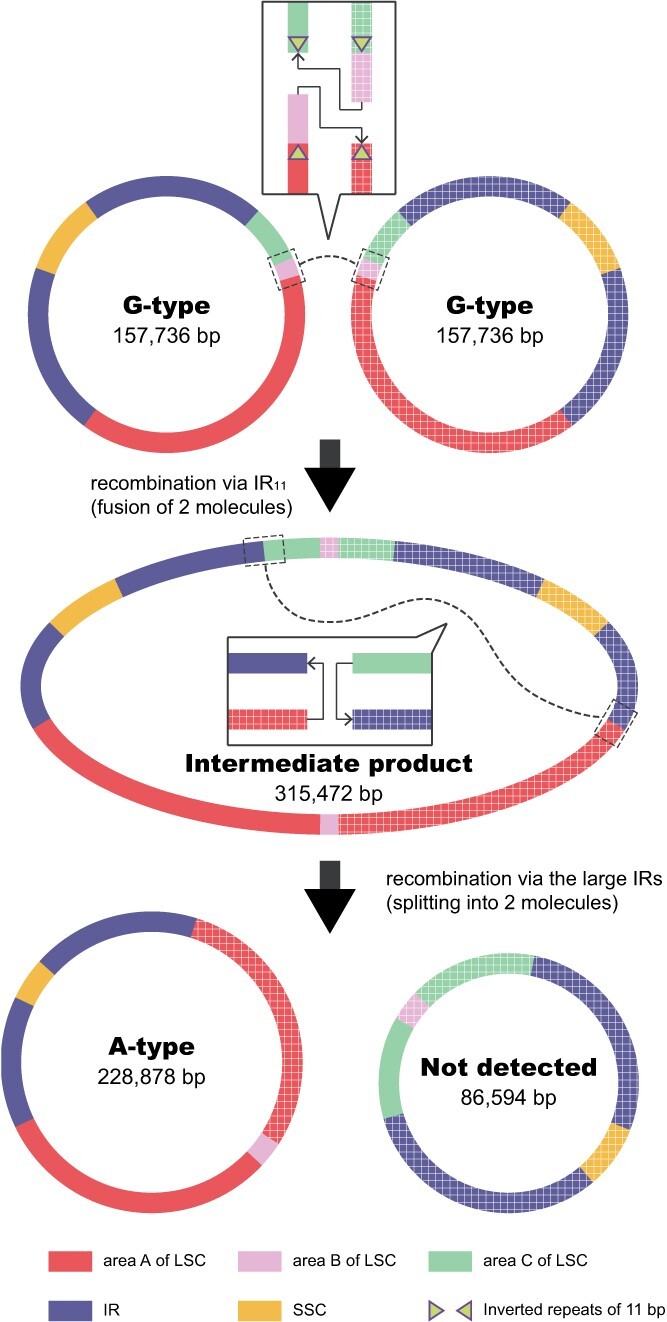
Schematic diagram showing the formation of the A-type plastome in *D. tasmanica* through intermolecular recombination of the G-type plastome. The first recombination mediated by the 11-bp IRs merges two G-type plastomes into a recombined intermediate product. The second recombination mediated by the large IRs splits the recombined intermediate product into an A-type plastome and a rearranged plastome (not detected). Solid lines and gridded lines represent sequences from two copies of the G-type plastome. Dotted lines and boxes indicate the positions of recombination. The recombination positions are magnified to show how the recombination proceeds.

Examination of the G-type plastome identified a pair of 11-bp IRs flanking the 92-bp area B in the LSC (Fig. 3). Hereafter we use the designation ‘IR_11_’ for the small IR to distinguish it from the large IR in the plastome. The sequences of IR_11_ were TTTTTTTTTTC and GAAAAAAAAAA. Given that IRs can mediate both intramolecular and intermolecular recombination in plastomes [26, 38, 73, 75, 76], we propose an intermolecular recombination model to explain the formation of the A-type plastome. As shown in Fig. 4, intermolecular recombination between two G-type plastomes mediated by IR_11_ could generate a hypothetical intermediate product, and further recombination at the large IR could generate two recombined circular molecules: one that contains a duplication of area A and loss of area B (corresponding to the A-type plastome), while the other has lost area A and duplicated area B. This latter recombined molecule, with area A deleted, was not detected in the albino and green sectors of the variegated individuals, based on both plastome assembly and mapping of Illumina reads to the recombined sequence. It is highly likely to be selectively eliminated because of inability to replicate or segregate vegetatively with so many genes being deleted.

In the G-type plastomes, we also tested whether the 92-bp area might undergo inversion mediated by intramolecular recombination at the flanking IR_11_ inverted repeat. However, mapping of Illumina reads to the hypothesized inversion conformation failed to detect the 92-bp inversion in leaves of green individuals.

### Plastomic heteroplasmy exists in both green and albino sectors of variegated individuals

The read depth (~1260 ×) is relatively even across the G-type plastome in the green individual (Supplementary Data Fig. S4). No A-type plastome was detected in leaves of the green individual based on Illumina read mapping. For green sectors of the two variegated individuals, we assessed the proportions of the G- and A-type plastomes using the depth information of area C based on its presence and absence in the two plastomes and of the SSC present in both plastomes as a single-copy region (see Materials and methods section). The average depths of the G- and A-type plastomes in one individual are 1198× and 876×, respectively, and those in the other individual are 270× and 297×, respectively (Supplementary Data Fig. S4). Although only the A-type plastome was assembled for albino sectors, the possibility of the presence of a low proportion of the G-type plastome cannot be excluded. Using Illumina read mapping, 3906× of the A-type plastome and 93× of the G-type plastome were detected in albino sectors of one individual, and 1774× and 39× for the other individual (Supplementary Data Fig. S4). This indicates that both green and albino sectors of the two variegated individuals have heteroplasmic plastomes, and that the G-type plastome exists at a very low frequency in albino sectors. The 92-bp area B shows a much lower average depth (2270× and 857×) in albino sectors of the two individuals, which may be caused by the formation of a hairpin secondary structure when this 92-bp segment is flanked by large IRs (including IR_11_ and the original LSC of the G-type plastome). It is well known that there is difficulty in sequencing regions with the ability to form a hairpin secondary structure. We also checked the original Illumina reads and found many read pairs at this position, with one incomplete read of only 35–98 bp in length. However, the depth of this area is not reduced in the green individual, which suggests little influence on sequencing of the unstable hairpin secondary structure when the 92-bp segment is flanked only by the small IR_11_.

### Gene loss in the A-type plastome and expressional changes of plastid genes are likely responsible for albinism in the leaf margins

**Figure 5:**
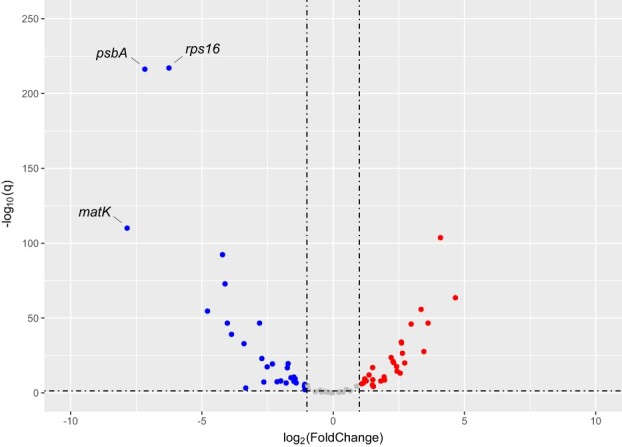
A volcano plot showing expressional difference in plastid genes between albino leaf sectors of the variegated plants and leaves of the green plants of *D. tasmanica*. Genes with significantly reduced and increased expression in the albino sectors are plotted in blue and red dots, respectively. The horizontal dashed line marks *Q* = 0.05.

The products of the three lost protein-coding genes and one tRNA gene (*matK*, *psbA*, *rps16*, and *trnK*) play important roles in photosynthesis, transcription, and translation (see Discussion section). This may cause the loss of photosynthesis, and thus albinism in the leaf margins of *D. tasmanica*. To further assess the influence of this structural variation on the expression of other plastid genes, we performed RNA-seq for the leaves of the green individuals, and green and albino sectors of the variegated individuals (Supplementary Data Table S2). Relative to the leaves of the green individuals, albino sectors of the variegated individuals showed significantly elevated expression in 28 genes and decreased expression in 29 other genes (Fig. 5, Supplementary Data Table S3). The three lost genes show the most dramatic decrease in expression; however, their expression levels do not reach zero, which presumably results from the low proportion of G-type plastomes retained in the sequenced albino sectors. Genes with reduced expression are mainly photosynthesis-related genes (*psa* and *psb*), suggesting an overall decrease of photosynthesis efficiency in albino sectors. In contrast, many genes related to transcription (*rpo*), translation (*rpl* and *rps*), ATP synthesis (*atp*), and photosynthetic regulation (*ndh*) show elevated expression in albino sectors, suggesting the maintenance of other plastid functions. Green sectors of the variegated individuals have an intermediate level of expression for all genes between the leaves of the green individuals and albino sectors of the variegated individuals (Supplementary Data Table S3).

### Albino tissues were observed in all leaf sectors of variegated individuals

**Figure 6:**
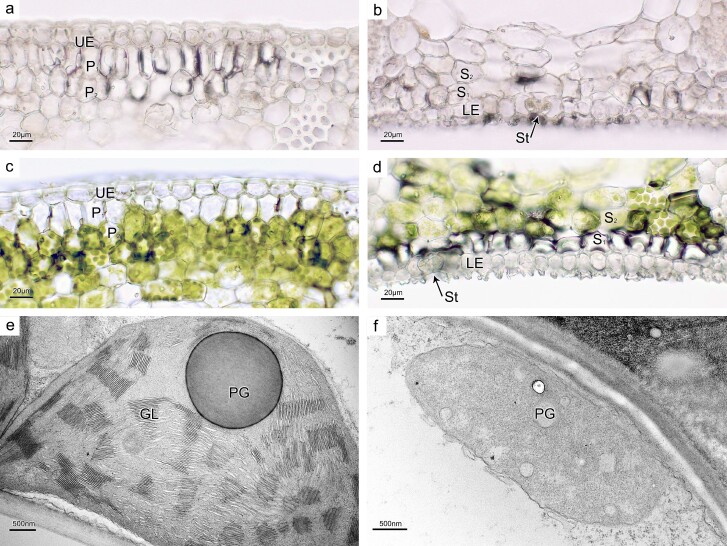
Leaf transverse sections of a variegated individual of *D. tasmanica* showing the distribution of albino mesophyll tissue (**a**–**d**) and transmission electron micrographs showing chloroplast ultrastructure in green and albino mesophyll cells (**e**, **f**). **a** Adaxial part of transverse section through the albino sector. **b** Abaxial part of transverse section through the albino sector. **c** Adaxial part of transverse section through the green sector, with one albino cell layer beneath the upper leaf epidermis. **d** Abaxial part of transverse section through the green sector, with one albino cell layer beneath the lower leaf epidermis. **e** A chloroplast in a green mesophyll cell of the green sector, with well-developed grana lamellae (GL). **f** A chloroplast in a mesophyll cell of the albino sector, without GL. UE, upper epidermis; LE, lower epidermis; P1, first layer of palisade mesophyll; P2, second layer of palisade mesophyll; S1, first layer of spongy mesophyll (first layer beneath the lower epidermis); S2, second layer of spongy mesophyll; St, stoma; PG, plastoglobule.

Microscopic examination of transverse sections indicated that mesophyll tissues in leaves of green individuals exhibited full pigmentation (Supplementary Data Fig. S5a, e, and f). In contrast, variegated individuals had albino tissue in all leaf sectors, including the albino, intermediate, and green sectors. All mesophyll tissues in the albino sector lacked pigmentation (Fig. 6a and b, Supplementary Data Fig. S5d). In the intermediate sector, albino mesophyll cells were observed in one to multiple layers directly beneath the upper leaf epidermis and in one layer beneath the lower leaf epidermis (Supplementary Data Fig. S5c, g, and h). One layer of albino cells was also present in the green sector, located beneath both leaf epidermal layers (Fig. 6c and d, Supplementary Data Fig. S5b). Despite a remarkable difference in the pigmentation of mesophyll tissues, the guard cells surrounding stomata on the lower leaf epidermis exhibited the same pigmentation (light yellowish green) in leaves of green individuals and in the green, intermediate, and albino sectors of variegated individuals (Fig. 6b and d, Supplementary Data Fig. S5f and h).

### Albino mesophyll cells had poorly developed chloroplasts lacking both grana and stroma lamellae

Mesophyll cells in leaves of green individuals contained fully developed chloroplasts with well-organized grana and stroma lamellae (Supplementary Data Fig. S6a and b). In contrast, mesophyll cells in the albino sector of variegated individuals had poorly developed chloroplasts lacking both grana and stroma lamellae (Fig. 6f, Supplementary Data Fig. S6c and d). Green mesophyll tissues of the intermediate and green sectors of variegated individuals had chloroplasts with similar ultrastructure to those in leaves of green individuals (Fig. 6e, Supplementary Data Figs S6e and f andS7c and d), while albino mesophyll tissues of the intermediate and green sectors of variegated individuals had chloroplasts comparable to those found in the albino sector (Supplementary Data Fig. S7a, b, e, and f). Chloroplasts found in the guard cells on the lower leaf epidermis of all tissues (leaves of green individuals, and albino, intermediate, and green sectors of variegated individuals) had similar ultrastructure, containing loosely arranged thylakoids and large starch granules (Supplementary Data Fig. S8).

## Discussion

### Intermolecular recombination leads to the unusual large plastome in variegated individuals of *D. tasmanica*

Expansion of plant plastomes is mainly due to IR expansion (e.g. [4, 10, 92, 98]), proliferation of AT-biased repeats in the non-coding regions [27] and LSC expansion [100]. Variegated individuals of *D. tasmanica* have the fourth largest plastome (228 878 bp), the longest IRs (105 226 bp), and the shortest LSC region (92 bp) in land plants yet sequenced. The extent of IR expansion and LSC contraction in the A-type plastome of variegated plants of *D. tasmanica* is unprecedented among land plants. The giant plastome in variegated plants of *D. tasmanica* is a consequence of substantial IR expansion into the LSC. Although the plastomes of *P. transvaalense* and *P. endlicherianum* are larger [92], their IRs (87 724 and 82 757 bp, respectively) are smaller than the A-type IRs of *D. tasmanica*. Different mechanisms have been proposed to explain IR expansions, such as gene conversion or double-strand DNA breaks [20, 89]. However, in *D. tasmanica* it appears that intermolecular recombination mediated by a pair of 11-bp IRs leads to substantial IR expansion. That the 92-bp area B and its flanking IR_11_ can form a stem-loop (hairpin) secondary structure, and the corresponding conformational changes around the 92 bp, including IR expansion and sequence deletion, is similar to the intermolecular recombination events reported in long-term cultured cell lines and albino plants regenerated by anther culture in rice [38].

Recombination in plant plastomes is well recognized, especially the well-known intramolecular flip-flop recombination mediated by the IR, which can create two structural conformations differing in the orientation of single-copy regions [68, 82, 90]. In addition to the large IR, other repeats can also mediate intra- or intermolecular recombination and generate inversions when repeats are inverted or deletions when they are direct [3, 25, 29, 36, 66, 96]. In many cases, short IRs in the plastomes apparently induce structural heteroplasmy. For example, a pair of small IRs (11 bp) could induce a 34-kb inversion, resulting in plastomic structural heteroplasmy in *Calocedrus macrolepis* [73]. Short direct and inverted repeats of 8–13 bp in *Oryza sativa* and 5–9 bp in *Triticum aestivum* and *Aegilops squarrosa* can mediate intramolecular recombination events in their plastomes [36, 37, 43, 66, 81]. Short IRs of 7–14 bp can also mediate intermolecular recombination in long-term cultured cell lines and albino plants regenerated by anther culture of rice [38]. Intramolecular recombination mediated by short IRs (<25 bp in length) can lead to both small inversions [3, 7, 25, 29, 37, 40–43] and large inversions [26, 28, 59, 73], based on the distance spanned by the repeats.

Considering that short direct and inverted repeats are not uncommon in plant plastomes, and that photosynthesis is not necessary for albino sectors, structural variation of this kind may not be rare in plastomes of variegated plants. Expanded sampling of various variegated plants may uncover more structural variations in their plastomes. It is expected that larger plastomes may exist in other variegated plants, if they possess such a kind of short IRs located closer to the LSC boundary to create larger IRs.

### Gene loss in the A-type plastome is likely responsible for albinism in the leaf margin

Variegation is common in plants and previous studies have identified spontaneous single-nucleotide mutations or single small insertions/deletions that are responsible for albinism in variegated plants [2, 23, 60, 70]. However, in *D. tasmanica* it appears that intermolecular recombination mediated by a pair of 11-bp IRs leads to a 7094-bp deletion and thus the appearance of albinism. In fact, albeit not being spontaneous, artificial introduction of plastome rearrangement by plastid-targeted forms of restriction endonucleases in *Arabidopsis* can cause leaf variegation [45, 83]. The deleted area in *D. tasmanica* contains four genes essential for photosynthesis (*psbA*), post-transcriptional processing (*matK*), and translation (*rps16* and *trnK*). Notably, *psbA* encodes photosystem II protein D1, which is one of the two core chlorophyll binding proteins for the reaction center of photosystem II [80]. It plays critical roles in maintaining conformational stability of the reaction center of photosystem II and electron transport [1, 64]. Therefore, loss of *psbA* may make cells in the leaf margins reduce and even lose the ability to photosynthesize. In autotrophic angiosperms, loss of *psbA* has not been reported yet, but transformed tobacco with deleted *psbA* showed reduced expression in genes of photosynthesis, energy metabolism, and chloroplast biogenesis [52].

Loss of transcription- and translation-related genes is also likely to disrupt plastid function. *matK* encodes a plastid intron maturase that aids excision of plastid group II introns of land plants [65, 101]. Without the essential enzyme in the plastome, some of the plastid proteins could not be made. A previous study showed that disruption of *matK* by a 19-bp insertion in its coding region is associated with chlorophyll deficiency in a variegation mutant of *C. japonica* [32]. *rps16* encodes ribosomal protein S16 and is needed for the function of the plastid translational apparatus. Although *rps16* has been lost from plastomes of some species, like *Medicago truncatula* and *Populus alba* [67, 78], the protein of nuclear-encoded *rps16* in these species can target their plastids to compensate for the loss [88]. *trnK* produces tRNA-Lys, the only plastid-encoded tRNA for lysine; loss of this tRNA would inhibit translation without supplementation of a tRNA-Lys from the cytosol. Collectively, loss of these four genes in the A-type plastome may cause the loss of photosynthesis, as proven by the dramatic reduction in expression of photosynthesis-related genes, and thus albinism in the leaf margins of *D. tasmanica.*

### How is the variegation pattern formed and maintained?

This unusual plastome conformation is not detected in green plants of this species, but it exists predominantly (~98%) in albino sectors, and occupies a considerable proportion (~43–52%) in green sectors of variegated plants. Thus, plastomic heteroplasmy exists not only within individual variegated plants but also within each of the green and albino sectors of variegated plants of this species. This is verified by our microscopic and TEM examination. Albino tissues were observed in all leaf sectors of variegated individuals, including albino, intermediate, and green sectors, and mesophyll cells in the albino tissues of variegated individuals had poorly developed chloroplasts lacking both grana and stroma lamellae. It is very likely that poorly developed chloroplasts lacking both grana and stroma lamellae carry the A-type plastome while fully developed chloroplasts with well-organized grana and stroma lamellae have the G-type plastome. Moreover, the roughly equal proportion of G and A type plastomes in green sectors is due to the presence of substantial albino tissue (Fig. 6c and d), and a low proportion of the G-type plastome in albino sectors likely results from normally developed chloroplasts in the guard cells of stomata (Fig. 6b, Supplementary Data Fig. S8), as shown by microscopic and ultrastructure observations.

Heteroplasmy in the shoot apical meristem (SAM) is thought to determine color patterns in variegated plants [16, 23, 24, 70, 87]. Similar to the situation in Figs 2A and4B of Frank and Chitwood [16], the A-type plastome in variegated plants of *D. tasmanica* likely arises in SAM initial cells, and propagates throughout the second meristem layer (L2) by periclinal division, creating a uniform, genetically distinct stratum of cells. This meristem layer, with the A-type plastome, gives rise to albinism along the leaf margins, while the inner corpus layer (L3), with the G-type plastome, generates green tissues within the leaf center. Some albino stripes in the central green sector may reflect invasion of cells in L2 to L3 of the SAM. With the G-type plastome in the outer meristem layer (L1), which forms the colorless epidermal cover, normally developed chloroplasts in the guard cells (belonging to the epidermal layer) in albino sectors of *D. tasmanica* are expected. Variegated plants of *D. tasmanica* are multiplied by vegetative propagation (tillering), so plastomic heteroplasmy and leaf variegation can be maintained from generation to generation.

## Materials and methods

### Sample collection, DNA extraction and sequencing

In variegated plants of *D. tasmanica*, green sectors are in the middle of the leaves and albino sectors are in the leaf margins, with some slim stripes in the middle of the leaves (Fig. 1). Fresh leaves of four individuals (two green individuals and two variegated individuals) of *D. tasmanica* were collected in the campus of Sun Yat-sen University, Guangzhou, China. For each of the two variegated individuals, albino sectors and green sectors were cut out separately. Samples of the two green individuals were designated as Ng1 and Ng2, and those of green and albino sectors of the two variegated individuals were designated as Vg1, Vg2, Va1, and Va2, respectively. DNA extraction from these leaf tissues was carried out with a HiPure Plant DNA Mini Kit (Magen, Guangzhou, China). Five shotgun DNA libraries (one each for Ng1, Vg1, Vg2, Va1, and Va2) with an insert size of 350 bp were constructed and then sequenced on an Illumina Novaseq 6000 platform, which generated paired-end reads of 150 bp with the nucleotide counts shown in Supplementary Data Table S1*.* In addition, one DNA library for Va1 with an insert size of 100 kb was constructed with the HLS HMW Library System (Sage Science) and the Ligation Sequencing 1D Kit (Oxford Nanopore Technologies, Oxford, UK) and then sequenced using Oxford Nanopore Technologies’ PromethION instrument, which generated 7.0-Gb reads with N50 length of 64 316 bp. RNA extraction from fresh leaf tissues was carried out with Trizol (Invitrogen, Carlsbad, CA, USA), and rRNAs were removed using the Ribo-Zero™ Magnetic Kit (Epicentre, Madison, WI, USA). Two biological replicates each were used for the leaves of green individuals and albino and green sectors of variegated individuals. Six transcriptome libraries with an insert size of 350 bp were constructed with the VAHTS^®^ Universal V8 RNA-seq Library Prep Kit (Vazyme, Nanjing, China) according to the manufacturer’s instructions and then sequenced on an Illumina Novaseq 6000 platform, which generated paired-end reads of 150 bp with the nucleotide counts shown in Supplementary Data Table S1.

### Plastome assembly and annotation

The raw Illumina reads were filtered with Trimmomatic 0.39 [5]. For each sample, we used clean Illumina reads for plastome assembly with GetOrganelle v1.7.5.1 [35] with the parameters: -F embplant_pt, −R 15, and -k 127. The assembly results were visualized in Bandage v0.8.1 [94] and plastome(s) were extracted from the assembly graphs based on high depth of coverage and blast results of the assembled contigs. The G-type plastome possesses a quadripartite structure typical of most angiosperms. For the A-type plastome, we used the assembled G-type plastome as the reference to extract plastid contigs from all assembled contigs using Blast in Bandage. One mitochondrial contig of plastid origin (MtPt) matching the G-type plastome was excluded based on its much lower depth. The A-type plastome has 11 contigs with sizes ranging from 200 to 57 090 bp (Supplementary Data Fig. S9). To confirm the rearrangement and the segment loss, Illumina and nanopore reads were mapped to the A-type plastome with BWA-mem [54] and Minimap2 [53], respectively. The mapping results were processed with SAMtools 1.13 [11] and then visulized in IGV_2.8.9 [84]. To confirm the whole A-type plastome structure, we drew dot plot diagrams with a 20-bp window size in Geneious 2022.0.2 software (http://www.geneious.com) to show the sequence collinearity between four randomly selected nanopore reads and the A-type plastome.

Protein-coding genes were annotated with GeSeq [85], and rRNA and tRNA genes were annotated with RNammer [46] and tRNAscan-SE (implemented in GeSeq) with the organelle option, respectively. The gene map of the plastome was plotted in OGDRAW [57].

### Assessment of plastomic heteroplasmy in the three tissues

We mapped Illumina reads to the plastomes to assess plastomic heteroplasmy in each of the three tissues. To test whether the A-type plastome is present in the green plant, we mapped Illumina reads of the green individual to the A-type plastome using BWA-mem [54] with default parameters. SAMtools 1.13 [11] was used to convert files and calculate depths, and IGV_2.8.9 [84] was used for visualization of read mapping. Depths of the A-type plastome can be calculated using the number of reads spanning over area B (see Results section for definition of areas A, B, and C) and its flanking 20 bp in area A at both ends. For albino and green sectors of the variegated individuals, we mapped their reads to the G-type plastome, and the depth of the G-type plastome can be calculated using the depth of area C. The depth of the A-type plastome can be calculated using the depth of the SSC minus the depth of area C. We also tested whether inversion of area B, which might be formed by intramolecular recombination mediated by the 11-bp IRs, exists in the green individual. The reversely complemented sequence of area B and the flanking 300-bp sequences of area B were used as the reference, and Illumina reads were then mapped to the reference. Numbers of reads spanning over the reversely complemented sequence of area B and the flanking 11-bp IRs were recorded. Moreover, we tested whether there was another hypothesized plastome conformation formed by intermolecular recombination in albino and green sectors of the variegated individuals. The sequence of area B and its flanking 300-bp sequence in area C at both ends was used as the reference, and Illumina reads were mapped to the reference using the same method. Numbers of reads spanning over the sequence of area B and the flanking 20 bp sequence at both ends were recorded.

### Analysis of plastid gene expression

Clean RNA-seq reads of each sample were mapped to the G-type plastome sequence with HISAT2 [71]. To facilitate calculation, one copy (IRa) of the IRs was excluded. The number of reads mapping to each plastid gene was calculated using HTSeq [72], with the following parameters: -a 10 -m union. Considering that the total expression level of all plastid genes may be different among different samples, which can influence the identification of differentially expressed genes if we use total reads mapping to all plastid genes for expression level normalization, we then used total read mapping to all transcripts in each sample for expression level normalization. To this end, we *de novo* assembled the transcriptome with clean RNA-seq reads of all six samples with Trinity 2.1.1 [22]. Redundant sequences in the assembled transcriptome were removed using Cd-hit 4.8.1 [55] with the parameter -c set to 0.95. Clean RNA-seq reads of each sample were then mapped to the non-redundant transcriptome sequences by HISAT2 [71], and the number of mapped reads was calculated with SAMtools 1.13 [11]. The TPM (transcripts per million reads) method was used to normalize the expression level for plastid genes. Differentially expressed plastid genes between the three tissues were identified using DESeq2 [58]. Differentially expressed genes with |log_2_FC| > 1 and Q value ≤0.05 were considered to be statistically significant. A volcano map showing the expressional differences of plastid genes was plotted with ggplot2 [18] in R.

### Microscopic characters of green and albino tissues

To investigate the distribution of green and albino tissues in leaves of *D. tasmanica*, we prepared freehand transverse sections of the leaves and examined their microscopic characters. The uppermost mature leaves were removed from both green and variegated individuals cultivated in the campus of Sun Yat-sen University and cleaned. Leaf sections were prepared for a green individual as well as albino, green, and intermediate sectors of a variegated individual using a double-edged blade, mounted on slides in distilled water, and then observed under a light microscope and photographed.

### Chloroplast ultrastructure of green and albino cells

To examine the difference of chloroplast ultrastructure between green and albino cells, we conducted TEM analysis of ultrathin leaf sections from a green individual as well as albino and green sectors of a variegated individual. Samples from a leaf of the green individual and green, intermediate, and albino sectors of a leaf of the variegated individual were cut into 3 × 3 mm pieces. All samples were vacuumized in 5% glutaraldehyde and 4% paraformaldehyde solution for 48 h, washed three times with 0.1 mmol/l PBS (pH = 7.2) buffer for 20 min, and then post-fixed with 2% OsO_4_ overnight followed by three more washes. The post-fixed samples were dehydrated in an ascending acetone series (20 min per step) and then infiltrated with Spurr’s resin. Ultrathin sections (70–100 nm) were prepared with a Leica EM UC7 ultramicrotome (Leica Microsystems, Wetzlar, Germany) and collected in copper grids. Sections were stained with uranyl acetate followed by three washes with distilled water and then in Reynold’s lead citrate followed by three more washes. Finally, samples were examined using a JEOL JEM-1400Flash Electron Microscope and photographed.

## Acknowledgements

We thank Shiquan Liang, Sanya Ke, and Qi Zeng for their help with plant sampling. This study was supported financially by the National Natural Science Foundation of China (31811530297).

## Author contributions

R.Z. designed the research, S.Z., Y.L., and K.M. performed the research and analyzed the data, and R.Z., S.Z., Y.L., and J.P.M. wrote the paper.

## Data availability

Raw Illumina DNA reads of five samples of *D. tasmanica* have been deposited in the NCBI SRA database (https://www.ncbi.nlm.nih.gov/sra) under accession numbers SRR25393521–SRR25393525. Raw nanopore reads of one sample of *D. tasmanica* have been deposited in the NCBI SRA database under accession number SRR26974411. Raw Illumina RNA-seq reads of six samples of *D. tasmanica* have been deposited in the NCBI SRA database under accession numbers SRR25409419–SRR25409423 and SRR25409519. Sequences and annotations of the G-type and A-type plastomes of *D. tasmanica* are available in NCBI GenBank (https://www.ncbi.nlm.nih.gov/genbank) under accession numbers OR345355 and OR345356.

## Conflict of interest

No conflict of interest declared.

## Supplementary data

Supplementary data is available at *Horticulture Research* online.

## Supplementary Material

Web_Material_uhae009
