# Supplementary material for: Leaf variegation caused by plastome structural variation: an example from Dianella tasmanica
Source: Hortic Res. 2024 Jan 10;11(3):uhae009. doi: 10.1093/hr/uhae009 (PMC10923649; doi:10.1093/hr/uhae009)
Supplement: Web_Material_uhae009 [file web_material_uhae009.zip › SupplementaryFigureS1-S9.pdf]

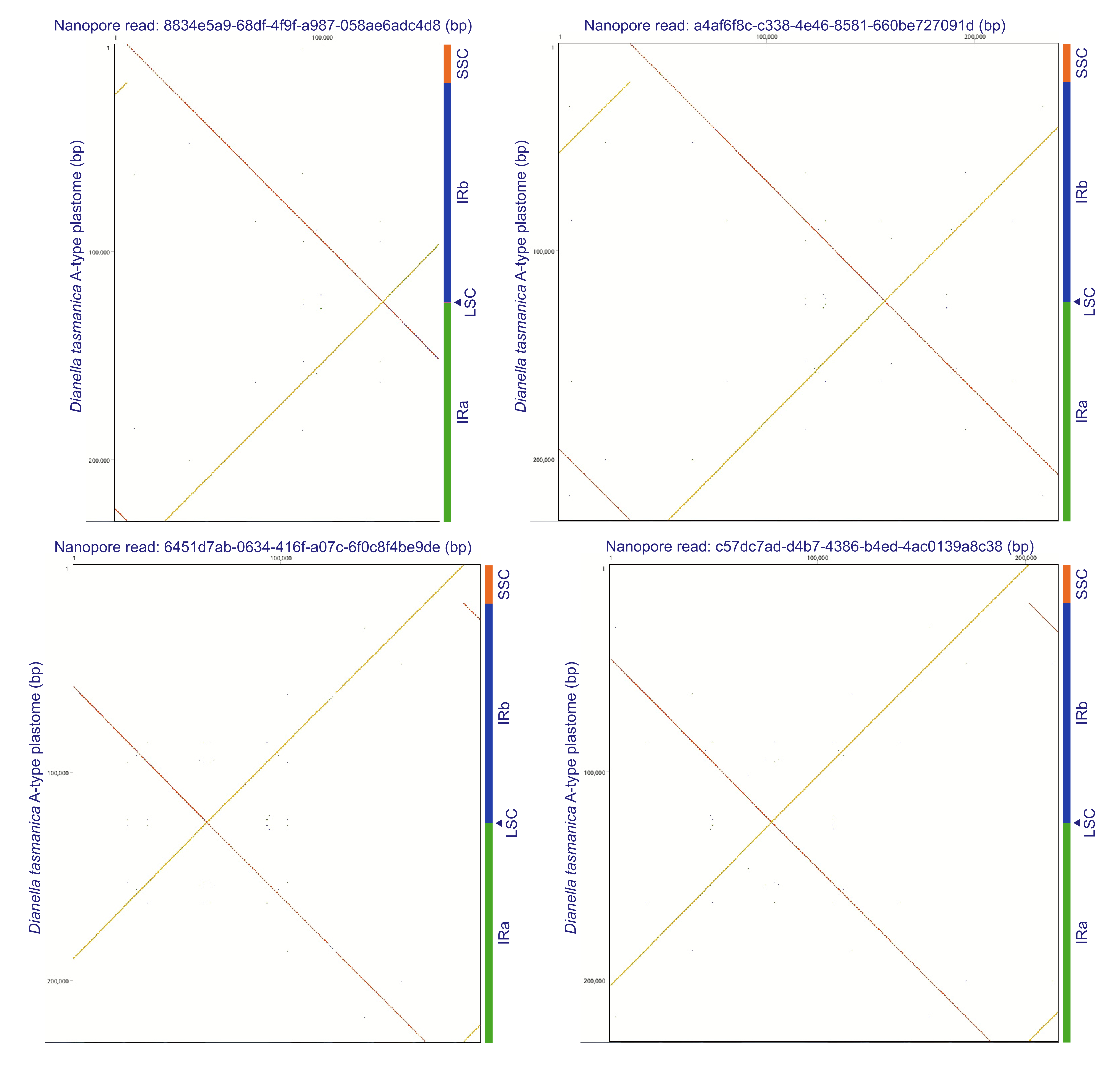

Figure S1. Structure of the whole A-type plastome of *Dianella tasmanica* supported by Nanopore reads. Sequence collinearity between four Nanopore reads and the A-type plastome was shown in dot plots (window size = 20 bp), using Geneious 2022.0.2 software (http://www.geneious.com). Red and yellow lines indicate strong collinearity from the forward and reverse direction, respectively. Only four Nanopore reads were shown here as examples because of space limitation.

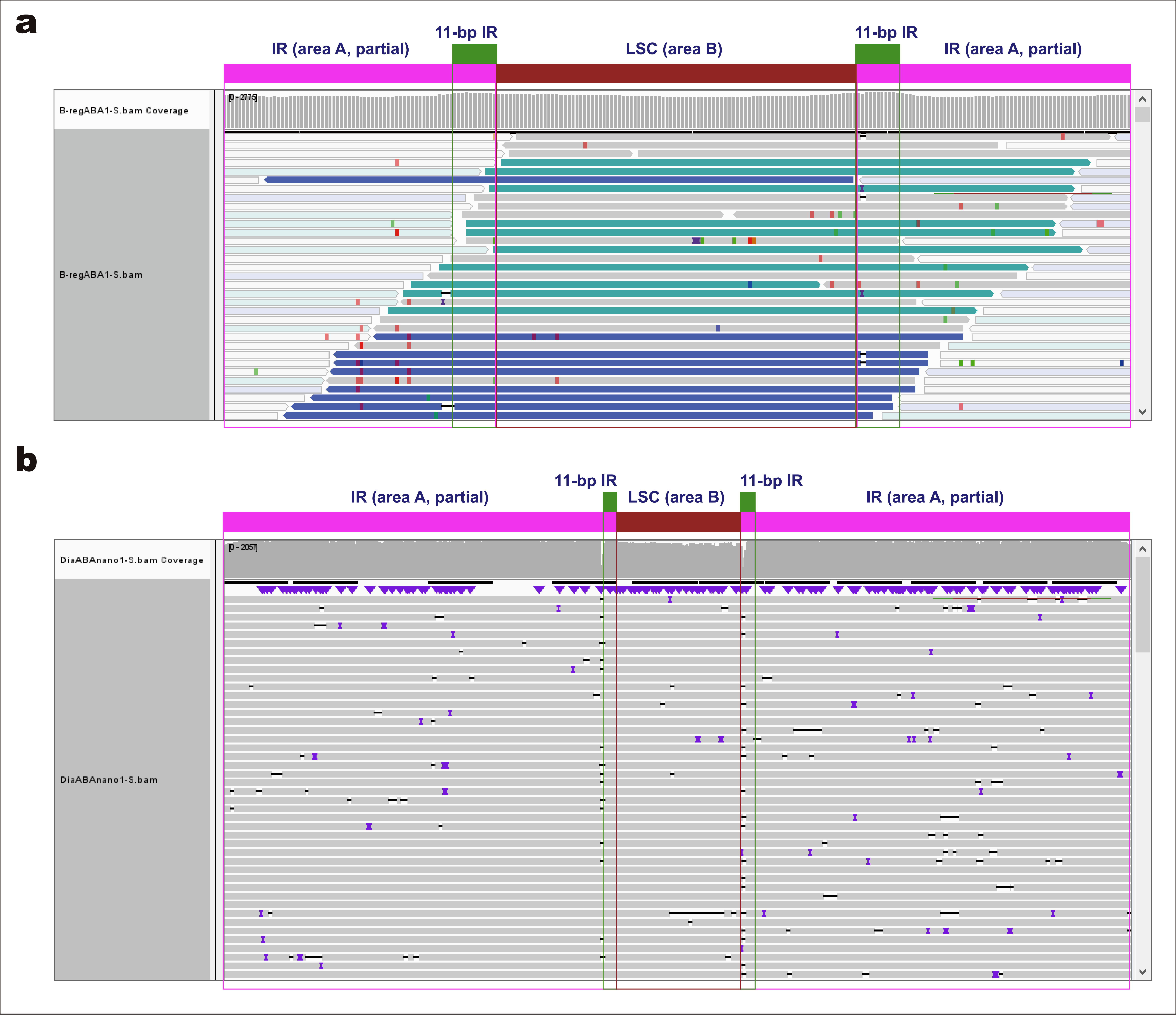

Figure S2. Structural rearrangement (the "A-B-A" structure) around the LSC (92 bp) of the A-type plastome of *Dianella tasmanica* supported by Illumina and Nanopore reads. Illumina and Nanopore reads of Va1 were mapped to the A-type plastome and the mapping results were visualized in IGV. Areas around the LSC of the A-type plastome, including B (= LSC), the 11-bp inverted repeats, A (belonging to a part of IR), were indicated with color bars. a: Illumina mapping result; b: Nanopore mapping result. Note that there exists sequencing errors for both Illumina and Nanopore reads.

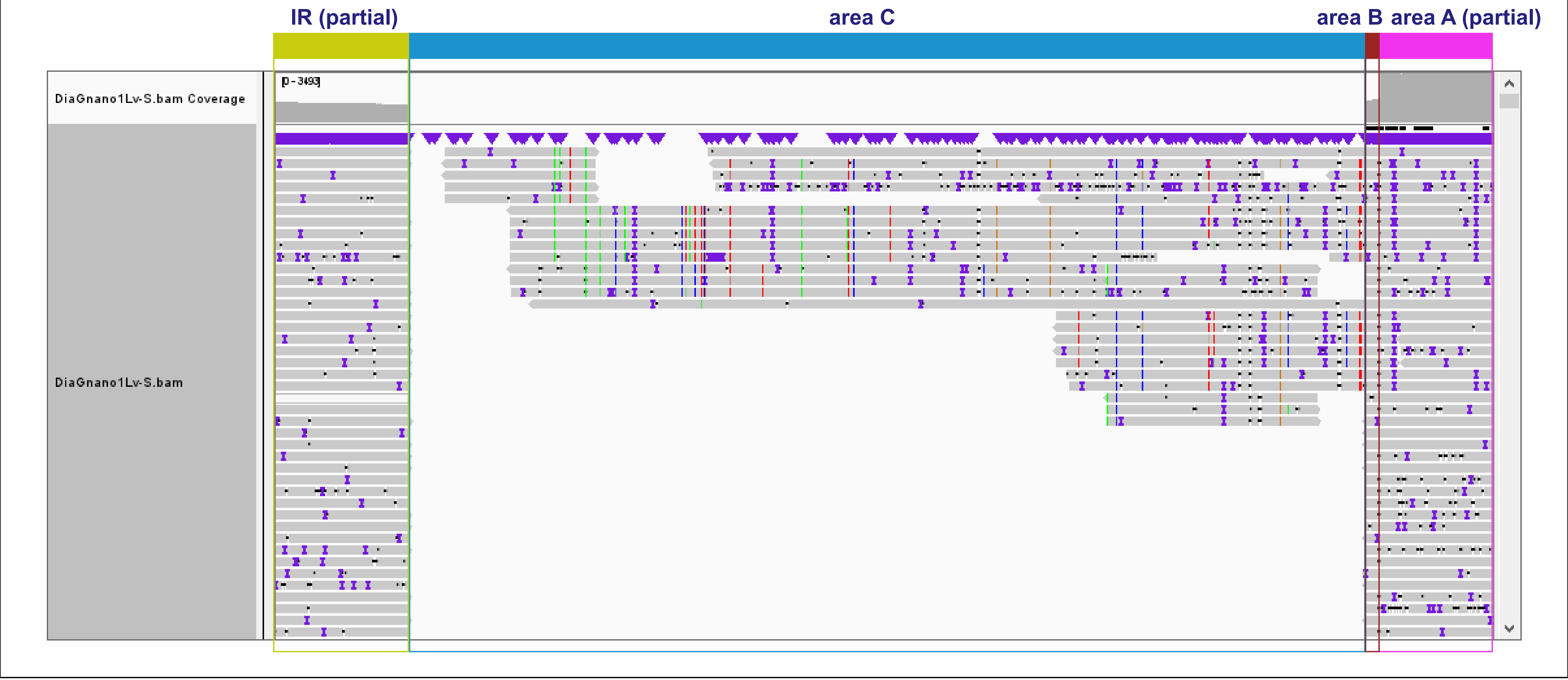

Figure S3. Loss of area C in the A-type plastome of *Dianella tasmanica* supported by Nanopore reads. Nanopore reads of Va1 were mapped to the G-type plastome which possesses area C. Regions of the G-type plastome were indicated with color bars. A small number of reads (~20×) that could be mapped to area C should result from plastomic heteroplasmy in the albino sectors, as described in the Results section. Area A has twice higher depth (~3000×) as much as area B and IR (~1500×), also supporting that it is a part of IR in the A-type plastome.

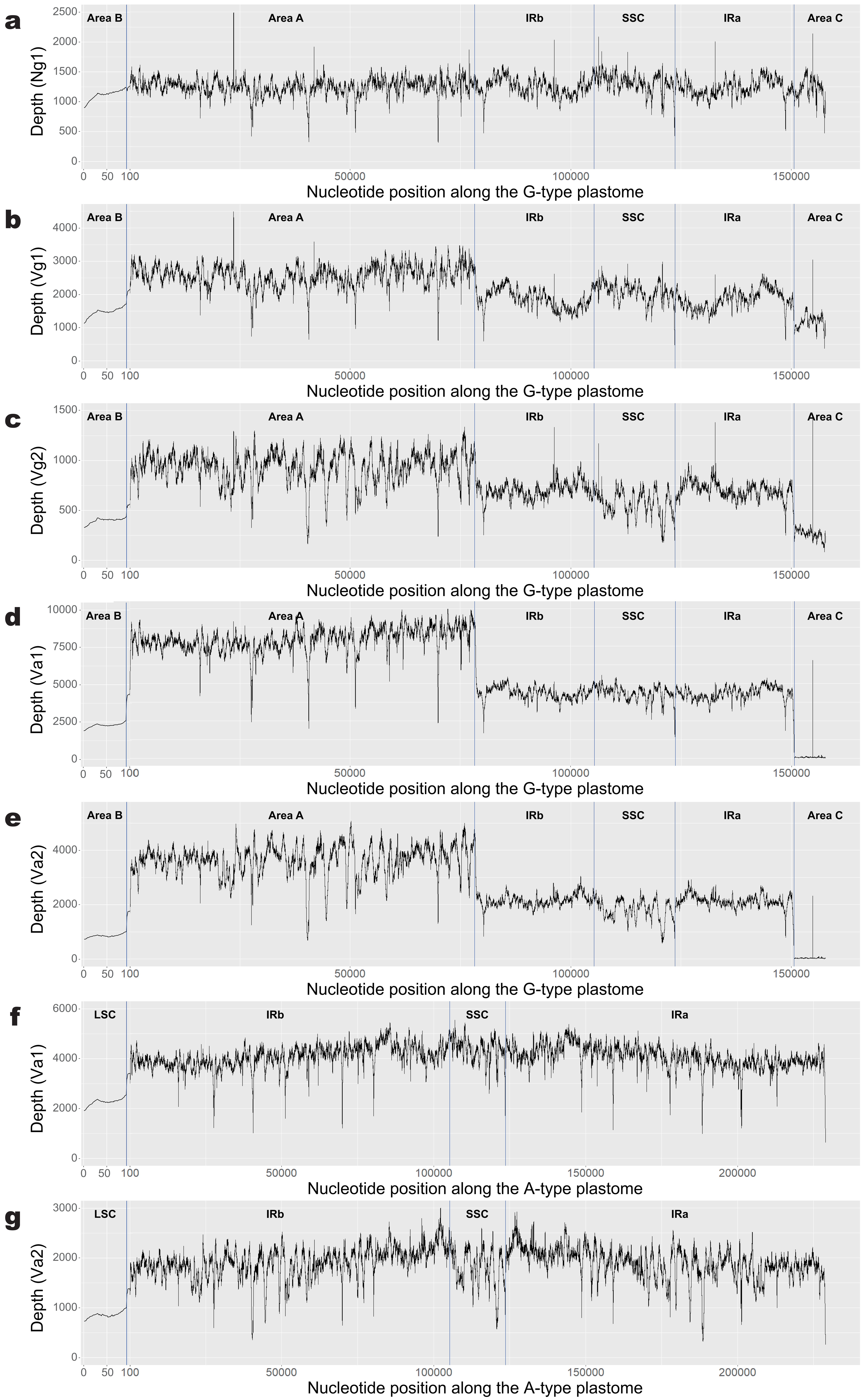

Figure S4. Sequencing depth of plastome for five samples of *Dianella tasmanica*. The five samples are Ng1, Vg1, Vg2, Va1 and Va2, in which Ng, Vg and Va stand for the green individual, green and albino sectors of the variegated individuals, respectively. To calculate sequencing depth, Illumina reads of the five samples (Ng1, Vg1, Vg2, Va1 and Va2) were mapped to the G- type plastome (panels a, b, c, d and e), and those of two samples (Va1 and Va2) were also mapped to the A-type plastome (panels f and g). Note that for the very short 92-bp area B in the G-type plastome (corresponding to LSC in the A-type plastome), the scale of X-coordinate was enlarged to make the depth information clear.

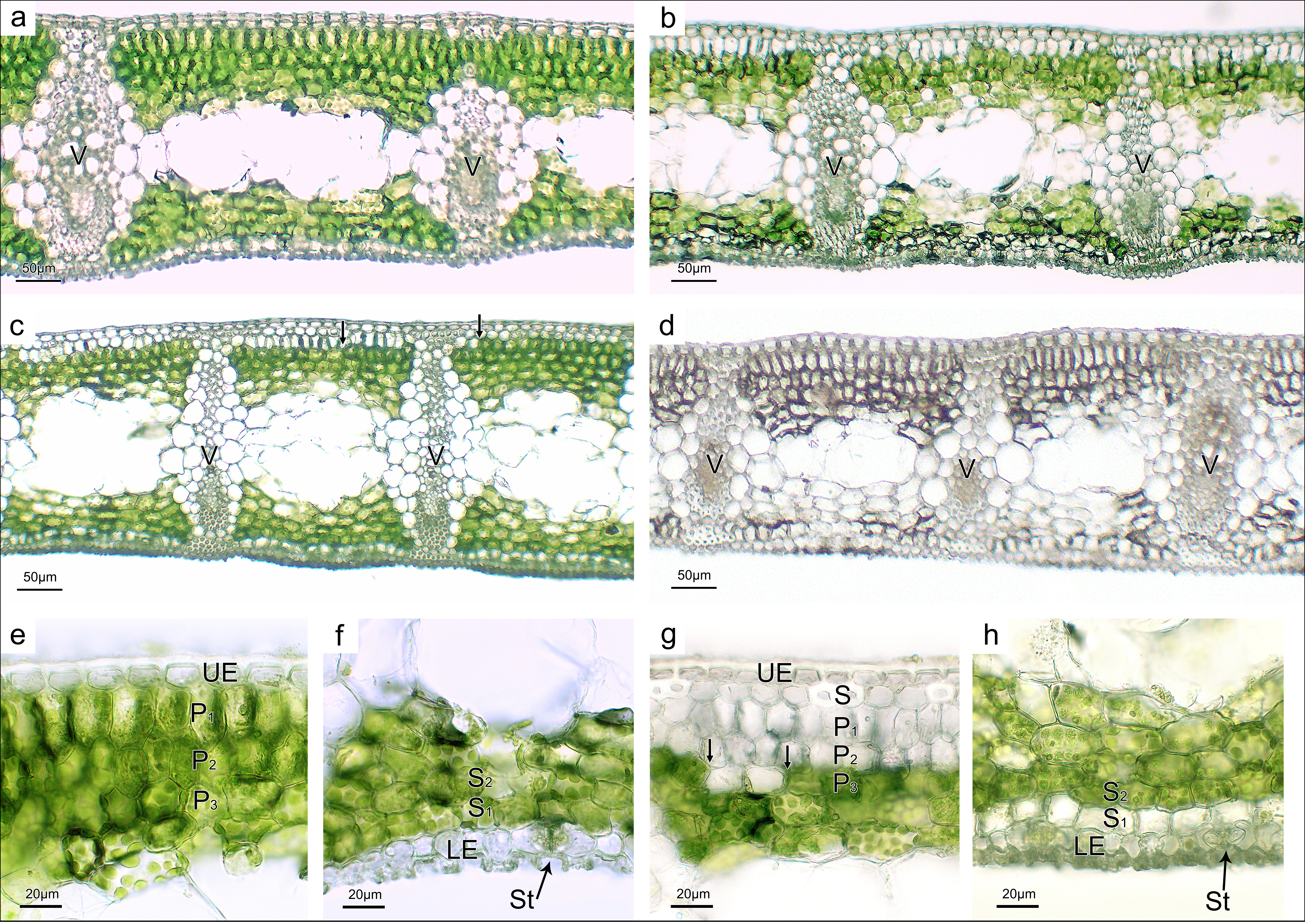

Figure S5. Leaf transverse sections of the green individual (a, e, f) and green (b), intermediate (c, g, h) and albino (d) sectors of the variegated individual of *Dianella tasmanica*, showing the distribution of green and albino mesophyll tissues. a) Section of the leaf of the green individual. b) Green sector of the variegated individual, with one albino cell layer beneath both upper and lower leaf epidermis. c) Intermediate sector of the variegated individual, sometimes with multiple albino cell layers beneath the upper leaf epidermis (arrows indicated the green-albino boundary). d) Albino sector of the variegated individual. e) Adaxial part of the transverse section of the leaf of the green individual. f) Abaxial part of the transverse section of the leaf of the green individual. g) Adaxial part of the transverse section through the intermediate sector. h) Abaxial part of the transverse section through the intermediate sector. V, vascular bundle; UE, upper epidermis; LE, lower epidermis; S, sclerenchymatous cell; P1, first layer of palisade mesophyll; P2, second layer of palisade mesophyll; P3, third layer of palisade mesophyll; S1, first layer of spongy mesophyll beneath the lower epidermis; S2, second layer of spongy mesophyll.

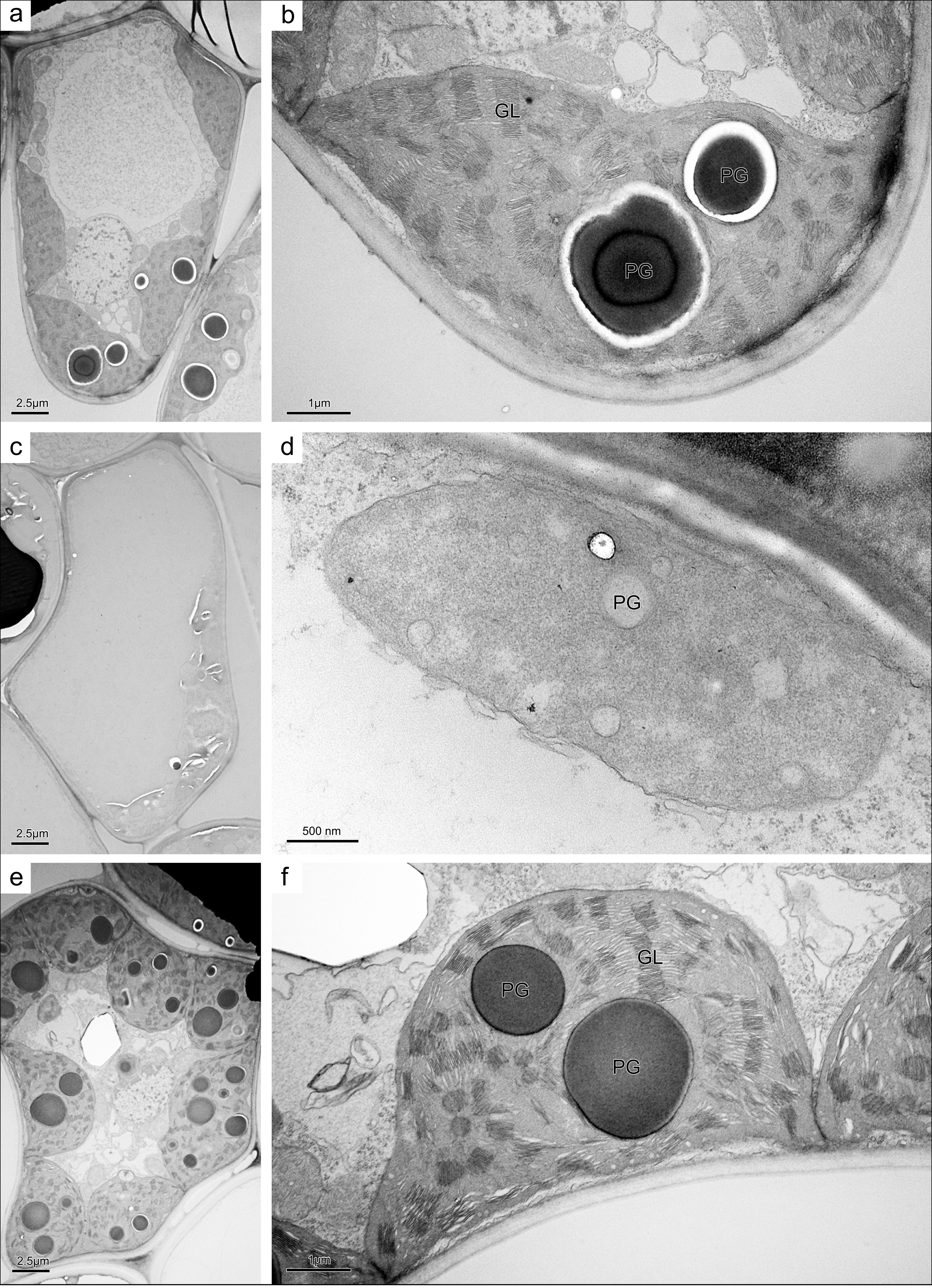

Figure S6. Transmission electron micrographs showing chloroplast morphology in the mesophyll cell of the green individual (a, b) and variegated individual (c, d, e, f) of *Dianella tasmanica*. a) A mesophyll cell in the green individual. b) A chloroplast in the mesophyll cell of the green individual. c) A mesophyll cell in the albino sector of the variegated individual. d) A chloroplast in the mesophyll cell of the albino sector. e) A green mesophyll cell in the intermediate sector. f) A chloroplast in the green mesophyll cell of the intermediate sector. PG, plastoglobules; GL, grana lamellae.

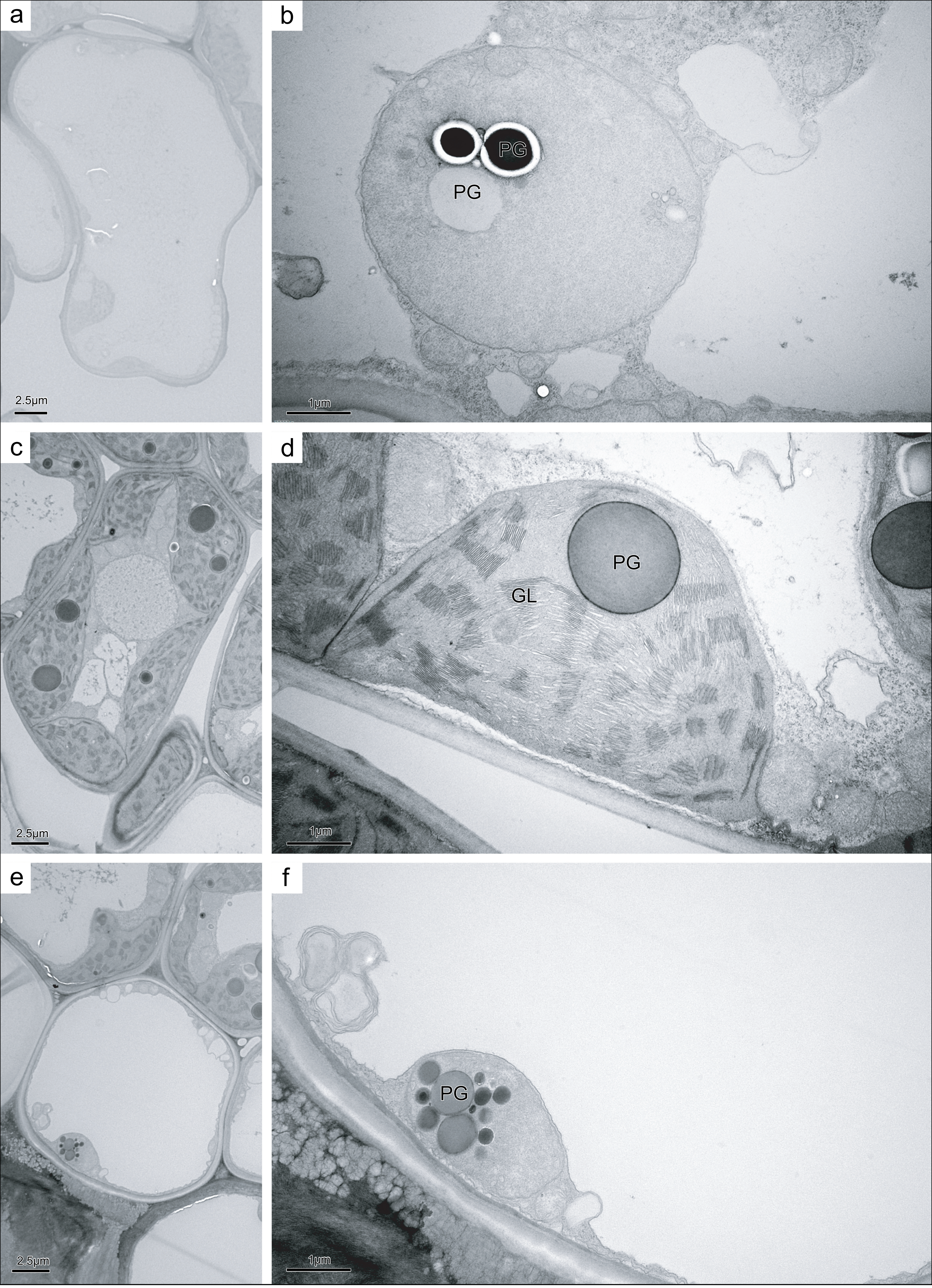

Figure S7. Transmission electron micrographs showing chloroplast morphology in the mesophyll cells of the variegated individual of *Dianella tasmanica*. a) An albino mesophyll cell of the intermediate sector. b) A chloroplast in the albino mesophyll cell of the intermediate sector. c) A green mesophyll cell of the green sector. d) A chloroplast in the green mesophyll cell of the green sector. e) An albino mesophyll cell of the green sector. f) A chloroplast in the albino mesophyll cell of the green sector. PG, plastoglobules; GL, grana lamellae.

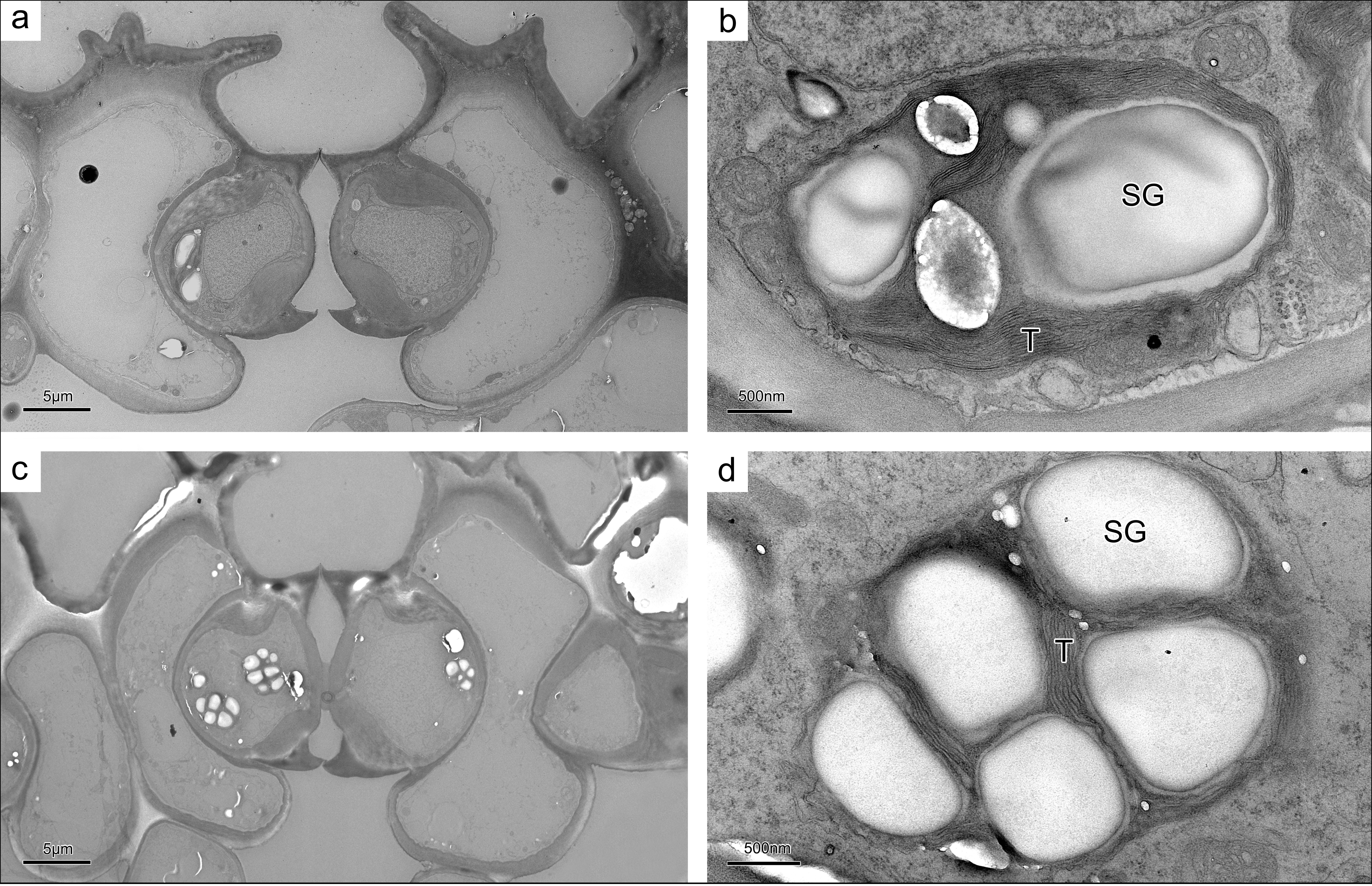

Figure S8. Transmission electron micrographs showing chloroplast morphology in the guard cells on lower leaf epidermis in green and albino sectors of the variegated individual of *Dianella tasmanica*. a) A stoma in the green sector. b) A chloroplast in a guard cell of the green sector. c) A stoma in the albino sector. d) A chloroplast in a guard cell of the albino sector. SG, starch granules; T, thylakoids.

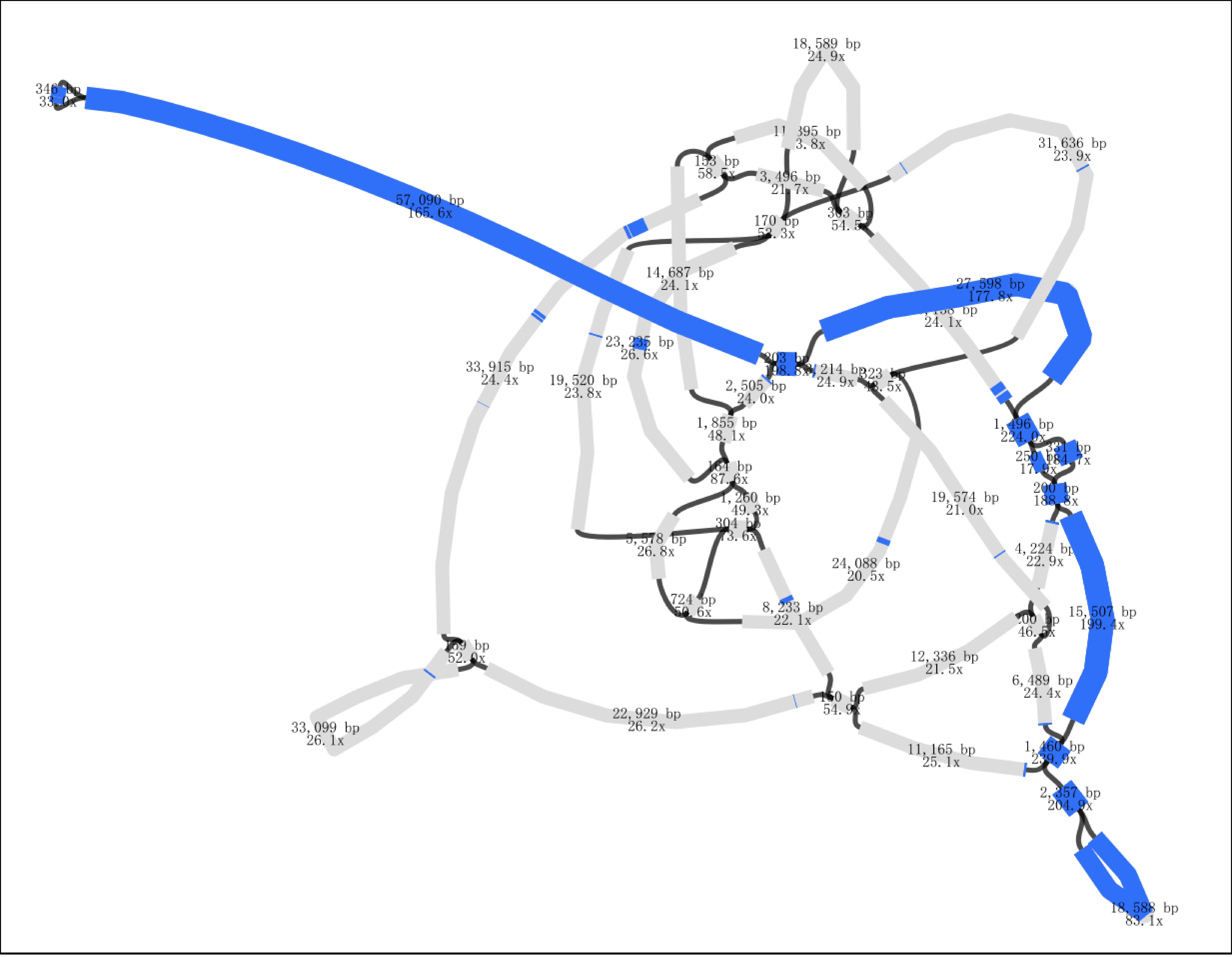

Figure S9. Assembly of the A-type plastome of *Dianella tasmanica*. The assembly was generated with GetOrganelle using Illumina reads of Va2 and visualized in Bandage. Note that this assembly contains contigs of both the plastome and the mitogenome due to the presence of mitochondrial sequences of plastid origin (MtPts). Contigs are connected with black lines, with the size and depth (calculated in GetOrganelle) of each contig indicated. Based on depth (much higher depth for plastid contigs) and sequence similarity to the G-type plastome based on Blast search in Bandage, 11 contigs are components of the A-type plastome. Blast hits to the G-type plastome were colored in blue.
